# The relationship between elderly nutritional risk index and short-term all-cause mortality in critically ill patients with cerebral injury: a retrospective cohort study from two cohorts

**DOI:** 10.3389/fnut.2025.1620364

**Published:** 2025-07-24

**Authors:** Zirong Gao, Xun Mo, Yi Ge, Yanhui Lu, Shanshan Yu

**Affiliations:** Department of Neuro Intensive Care Unit, The Affiliated Jinyang Hospital of Guizhou Medical University, Guiyang, China

**Keywords:** GNRI, cerebral injury, critically ill, mortality, MIMIC

## Abstract

**Background:**

The Geriatric Nutritional Risk Index (GNRI) is a simple and objective tool for assessing the risk of malnutrition, with potential practicality in critically ill patients with cerebral injury (CIPCI). However, the current evidence is still limited.

**Method:**

Patients diagnosed with cerebral injury were retrospectively retrieved from the Medical Information Marketplace for Intensive Care (MIMIC-IV) and the Affiliated Jinyang Hospital of Guizhou Medical University. Various statistical methods, including restricted cubic spline regression (RCS) regression, multivariate logistic regression, subgroup analyses, and KM survival curves, were used to examine the association between the GNRI index and short-term adverse outcomes in CIPCI. Finally, we used LASSO-COX regression and multifactorial COX regression to develop risk prediction models and assessed the validity of the risk models using subject work characteristics (ROC) curves, area under the ROC (AUC).

**Result:**

The final 1,244 CIPCIs were included in the analysis, of which the 28-day ICU/in-hospital mortality rates were 23.5 and 27.18%, respectively. In the fully adjusted model, continuous GNRI and GNRI category were significantly associated with 28-day ICU/in-hospital mortality, with HRs of, respectively, continuous GNRI: 0.97 (0.96–0.99)/0.97 (0.96–0.99) and [moderate nutritional risk vs. no nutritional risk: 1.66 (1.01–2.74) and 2.03 (1.26–3.28), higher nutritional risk vs. no nutritional risk: 1.79 (1.08–2.97)/2.11 (1.30–3.42)]. Meanwhile, the RCS showed an inverse linear association between continuous GNRI and both 28-day ICU/hospitalization mortality, and this association remained consistent across most subgroups. In addition, the results of the ROC curves showed that the risk model constructed in this study could identify highly-risk CIPCI more effectively than the traditional critical care score. Finally, all results have been further confirmed in the external queue.

**Conclusion:**

This study identifies that GNRI is negatively associated with short-term mortality in critically ill patients with cerebral injury and provides a simple and effective tool for risk stratification, allowing clinicians to identify at-risk individuals and provide timely intervention.

## Background

Neurological disorders currently represent the primary cause of global disability. Encephalopathy denotes an altered state of consciousness resulting from cerebral dysfunction, which may originate from diffuse or focal brain injury (1, 2). Given its substantial morbidity and mortality burden, brain injury commands significant clinical attention (3). Common neurological conditions include cerebral infarction, spontaneous subarachnoid hemorrhage, metabolic or toxic encephalopathies, traumatic brain injury, and other cranial pathologies (1–3).

Patients’ nutritional status significantly influences the incidence and prognosis of cerebral injury (4, 5). Multiple studies have examined the relationship between nutritional indicators and severe cerebral injury, highlighting the critical importance of early nutritional assessment and intervention (6, 7). While dozens of nutritional screening tools have been proposed, their clinical utility remains limited (8, 9). Consequently, no tool currently serves as the gold standard for identifying malnutrition risk. This gap underscores the need for a rapid, simple, and objective screening method to enable clinicians to effectively assess malnutrition risk in critically ill patients with cerebral injury (CIPCI) admitted to intensive care units (ICUs).

The Geriatric Nutritional Risk Index (GNRI) is a validated tool designed to assess the nutritional status of older adults. It calculates nutritional risk using only serum albumin levels and body mass index (BMI), offering advantages in simplicity and efficiency (10). Previous studies have demonstrated that low GNRI scores correlate with poor clinical outcomes in critical conditions such as heart failure, acute coronary syndrome, and sepsis (11–14). However, evidence remains limited regarding its association with outcomes in CIPCI. Therefore, this study aimed to investigate whether GNRI scores could predict clinical outcomes in this high-risk neurological population and further develop a risk proportion model with good predictive performance.

## Method and population

### Data source

This was a multicenter retrospective study using data from 2 cohorts. The study was approved by the Ethics Committee of the Affiliated Jinyang Hospital of Guizhou Medical University (Ethical approval number: 2-JY2025). The internal cohort was derived from the Medical Information Mart for Intensive Care IV (MIMIC-IV v3.1) (15): a publicly accessible, open-source repository curated by the Massachusetts Institute of Technology (MIT) comprising 247,366 individuals and 196,527 adults admitted to Beth Israel Deaconess Medical Center between 2008 and 2019. This database was accessed according to an approved protocol, with an Institutional Review Board (IRB) waiver due to retrospective analysis of de-identified data. The external cohort was obtained from patients with cerebral critical illness who were admitted to the Neurological Intensive Care Unit of Jinyang Hospital of Guizhou Medical University from January 2022–January 2025.

### Study population

This study focused on critically ill neurological patients admitted for the first time to a general intensive care unit (ICU). Diagnoses were classified using the International Classification of Diseases, 9th and 10th revisions (ICD-9/10), including traumatic brain injury, ischemic stroke, intracerebral hemorrhage, subarachnoid hemorrhage, intracranial infections, and related disorders. Inclusion criteria were: (1) age ≥18 years; (2) survival duration and ICU length of stay ≥24 h; (3) availability of complete anthropometric (weight, height) and serum albumin data. For patients with multiple ICU admissions, only data from their first admission were analyzed. Clinical data were collected within the first 24 h following ICU admission.

### Variable extraction

Extraction was performed using PostgreSQL (v13.7.2) and Navicat Premium (v16.0) with structured query language (SQL). Extracted variables were categorized into six groups:1. Demographics: Age, sex, weight, height, BMI.2. Comorbidities: Hypertension, acute kidney injury (AKI), chronic kidney disease (CKD), heart failure (HF), diabetes, chronic obstructive pulmonary disease (COPD) 0.3. Vital signs: Respiratory rate (RR), heart rate (HR), non-invasive blood pressure (mean: NIBP-M, systolic: NIBP-S, diastolic: NIBP-D) 0.4. Laboratory parameters: Red blood cell count (RBC), white blood cell count (WBC), hemoglobin (Hb), platelet count (PLT), red cell distribution width (RDW), hematocrit (HCT), lactate (Lac), serum sodium (Na), potassium (K), chloride (Cl), creatinine (Cr), blood urea nitrogen (BUN), glucose, international normalized ratio (INR), prothrombin time (PT), activated partial thromboplastin time (PTT), alanine aminotransferase (ALT), aspartate aminotransferase (AST), total bilirubin (TB), partial pressure of oxygen (PO₂), partial pressure of carbon dioxide (PCO₂)0.5. Disease severity scores: Acute Physiology Score III (APS III), Simplified Acute Physiology Score II (SAPS-II), Oxford Acute Severity of Illness Score (OASIS), and Sequential Organ Failure Assessment (SOFA) 0.6. Therapies: Vasopressors (VP), mechanical ventilation (MV), continuous renal replacement therapy (CRRT).

### Definitions of nutritional status and endpoints

Patients were stratified into four nutritional risk groups based on GNRI scores (10): No nutritional risk (GNRI ≥98), Low risk (92 ≤ GNRI <98), Moderate risk (82 ≤ GNRI <92), and High risk (GNRI <82). GNRI was calculated as: GNRI = [14.89 × serum albumin (g/dL)] + [41.7 × (actual BMI/ideal BMI)], with the ideal BMI defined as 22 kg/m^2^ (16, 17). The primary endpoint was 28-day ICU mortality, and the secondary endpoint was 28-day hospital all-cause mortality.

### Risk prediction modeling and validation

All patients were randomly allocated to training and validation cohorts in a 7:3 ratio. In the training cohort, we performed LASSO regression using the R package “*glmnet*” with the optimal lambda (*λ*) value to identify key variables strongly associated with the primary endpoint (28-day ICU mortality). Subsequently, a multivariable Cox regression model was applied to select prognostically significant factors for constructing the predictive model, which was visualized as a nomogram. Model performance was evaluated using receiver operating characteristic (ROC) curves and the area under the curve (AUC).

### Statistical analysis

Continuous variables are presented as mean ± standard deviation (SD) or median with interquartile range (IQR). Group comparisons were performed using t-tests, ANOVA, Mann–Whitney U tests, or Kruskal-Wallis tests, as appropriate. Categorical variables are expressed as numbers (percentages), and differences between groups were analyzed using Pearson’s chi-square or Fisher’s exact tests. Cox proportional hazard regression models were used to estimate hazard ratios (HRs) with 95% confidence intervals (95% CIs). Three models were constructed: Model 1 (unadjusted); Model 2 (adjusted for age, gender, Race, BMI); Model 3 (further adjusted for comorbidities, heart rate, non-invasive systolic blood pressure, vasopressor use, mechanical ventilation, continuous renal replacement therapy, and laboratory parameters differing between survivors and non-survivors; see Supplementary Tables S1–S3). Trend tests and variance inflation factors (VIFs) were assessed to evaluate multicollinearity (Supplementary materials). Restricted cubic splines (RCS) with four knots (5th, 35th, 65th, and 95th percentiles) were applied to explore potential nonlinear relationships between GNRI and outcomes. Subgroup analyses were conducted for predefined strata: age (>65 vs. ≤65 years), BMI (≤30 vs. >30 kg/m^2^), sex, hypertension, acute kidney injury (AKI), chronic kidney disease (CKD), and COPD. All analyses were performed using R Statistical Software (v4.2.2). A two-sided *p*-value <0.05 was considered statistically significant.

## Results

### Baseline data for patients with different nutritional status

A total of 1,224 critically ill neurological patients were included based on the eligibility criteria (Table 1). The cohort had a median age of 67 years (IQR: 55–79), with 763 males (62.3%). Stratified by GNRI: 382 patients (31.2%) in the high-risk group, 497 (40.6%) moderate-risk, 194 (15.9%) low-risk, and 151 (12.3%) no-risk. Baseline characteristics stratified by GNRI are shown in Table 1. Patients with nutritional risk (lower GNRI scores) tended to be older. Compared to the no-risk group, high-risk patients showed elevated levels of heart rate, respiratory rate, RDW, WBC, albumin, potassium, lactate, PCO₂, chloride, PTT, creatinine, and urea nitrogen, but lower hemoglobin, red blood cell count, hematocrit, anion gap, and blood pressure. Comorbidities including hypertension, AKI, CKD, heart failure, and COPD were more prevalent in high-risk groups. Clinical severity scores increased significantly with lower GNRI. The high-risk group exhibited substantially higher mortality than the no-risk group: 28-day ICU mortality (31.2% vs. 12.6%, *p* < 0.001) and overall ICU mortality (35.2% vs. 13.2%, *p* < 0.001).

**Table 1:** Summary descriptives table by groups of GNRI group.

	ALL	No	Low	Moderate	High	*p*-value
	N = 1,224	N = 151	N = 194	N = 497	N = 382	
GNRI	86.4 [80.4;92.3]	101 [98.3;104]	93.8 [92.3;95.3]	87.9 [84.9;89.3]	77.4 [74.1;80.4]	<0.001
Age	67.0 [55.0;79.0]	63.0 [51.0;71.0]	65.0 [52.2;75.8]	69.0 [56.0;80.0]	67.0 [56.0;79.8]	<0.001
Gender:						0.083
F	461 (37.7%)	44(29.1%)	76(39.2%)	201 (40.4%)	140 (36.6%)	
M	763 (62.3%)	107 (70.9%)	118 (60.8%)	296 (59.6%)	242 (63.4%)	
Race:						0.407
No	525 (42.9%)	69 (45.7%)	88 (45.4%)	199 (40.0%)	169 (44.2%)	
Yes	699 (57.1%)	82 (54.3%)	106 (54.6%)	298 (60.0%)	213 (55.8%)	
BMI	27.3 [24.0;31.9]	28.0 [25.0;32.2]	28.6 [25.0;33.3]	27.4 [23.8;31.9]	26.8 [23.0;30.8]	<0.001
Hypertension:						<0.001
No	668 (54.6%)	62 (41.1%)	98 (50.5%)	264 (53.1%)	244 (63.9%)	
Yes	556 (45.4%)	89 (58.9%)	96 (49.5%)	233 (46.9%)	138 (36.1%)	
AKI:						<0.001
No	670 (54.7%)	102 (67.5%)	123 (63.4%)	280 (56.3%)	165 (43.2%)	
Yes	554 (45.3%)	49 (32.5%)	71 (36.6%)	217 (43.7%)	217 (56.8%)	
CKD:						0.003
No	1,004 (82.0%)	134 (88.7%)	168 (86.6%)	408 (82.1%)	294 (77.0%)	
Yes	220 (18.0%)	17 (11.3%)	26 (13.4%)	89 (17.9%)	88 (23.0%)	
Diabetes:						0.398
No	853 (69.7%)	108 (71.5%)	144 (74.2%)	342 (68.8%)	259 (67.8%)	
Yes	371 (30.3%)	43 (28.5%)	50 (25.8%)	155 (31.2%)	123 (32.2%)	
HF:						0.018
No	882 (72.1%)	123 (81.5%)	144 (74.2%)	341 (68.6%)	274 (71.7%)	
Yes	342 (27.9%)	28 (18.5%)	50 (25.8%)	156 (31.4%)	108 (28.3%)	
COPD:						0.044
No	1,022 (83.5%)	136 (90.1%)	166 (85.6%)	413 (83.1%)	307 (80.4%)	
Yes	202 (16.5%)	15 (9.93%)	28 (14.4%)	84 (16.9%)	75 (19.6%)	
SOFA	6.00 [3.00;9.00]	4.00 [2.00;6.00]	5.00 [3.00;7.75]	5.00 [3.00;8.00]	7.00 [5.00;10.0]	<0.001
APSII	49.0 [36.0;66.0]	40.0 [29.0;55.0]	43.0 [33.2;58.0]	47.0 [35.0;63.0]	58.0 [46.0;75.8]	<0.001
SAPII	40.0 [31.0;51.0]	32.0 [25.0;42.5]	38.0 [29.0;47.0]	40.0 [31.0;50.0]	46.0 [37.0;56.0]	<0.001
OASIS	36.0 [31.0;42.0]	31.0 [26.0;38.5]	34.0 [28.2;41.0]	36.0 [31.0;42.0]	40.0 [34.0;44.0]	<0.001
GCS	15.0 [11.0;15.0]	14.0 [10.0;15.0]	15.0 [10.0;15.0]	15.0 [11.0;15.0]	15.0 [13.0;15.0]	0.001
HR	87.0 [75.0;101]	83.0 [74.0;95.0]	89.0 [73.0;99.0]	85.0 [72.0;98.0]	91.5 [79.0;108]	<0.001
RR	19.0 [16.0;22.0]	18.0 [15.0;21.0]	18.0 [15.0;22.0]	19.0 [15.0;22.0]	20.0 [16.0;23.0]	0.004
NBPS	124 [105;143]	139 [120;154]	130 [112;146]	126 [104;143]	115 [97.2;131]	<0.001
NBPD	68.0 [57.0;81.0]	74.0 [61.0;87.0]	71.0 [59.2;83.0]	68.0 [57.0;83.0]	64.0 [54.2;75.0]	<0.001
NBPM	82.0 [70.0;95.0]	90.0 [77.0;104]	86.0 [73.2;95.0]	82.0 [70.0;96.0]	77.4 [66.0;89.8]	<0.001
HCT	33.5 [28.7;38.2]	36.5 [32.8;40.7]	35.5 [31.5;39.9]	33.5 [28.8;38.0]	31.2 [27.2;35.5]	<0.001
Hb	11.1 [9.40;12.7]	12.6 [10.9;13.6]	11.9 [10.5;13.4]	11.0 [9.30;12.7]	10.0 [8.75;11.7]	<0.001
PLT	192 [137;255]	205 [153;260]	192 [145;251]	190 [140;249]	186 [118;260]	0.188
RDW	14.3 [13.4;15.6]	13.8 [13.1;14.9]	13.8 [13.2;15.1]	14.4 [13.5;15.6]	14.7 [13.7;16.2]	<0.001
RBC	3.67 [3.14;4.22]	4.07 [3.54;4.50]	3.92 [3.43;4.44]	3.68 [3.16;4.21]	3.38 [2.94;3.88]	<0.001
WBC	12.3 [8.88;16.2]	11.6 [8.10;14.9]	12.6 [9.40;15.7]	12.3 [8.80;16.4]	12.6 [8.95;17.1]	0.188
ALB	3.10 [2.70;3.50]	4.00 [3.85;4.20]	3.50 [3.42;3.60]	3.10 [3.00;3.20]	2.50 [2.20;2.70]	<0.001
AG	15.0 [12.0;17.0]	15.0 [13.0;18.0]	15.0 [12.0;17.0]	14.0 [12.0;17.0]	14.0 [12.0;18.0]	0.192
Glu	142 [115;183]	145 [116;180]	138 [116;181]	142 [116;185]	144 [113;183]	0.955
K	4.00 [3.70;4.50]	4.00 [3.70;4.30]	4.00 [3.60;4.50]	4.10 [3.70;4.60]	4.10 [3.80;4.70]	0.016
Na	139 [137;142]	139 [137;141]	139 [137;142]	139 [137;142]	140 [136;143]	0.253
CL	105 [101;109]	104 [100;107]	105 [101;108]	105 [101;109]	107 [102;111]	<0.001
LAC	1.90 [1.30;3.00]	1.80 [1.20;2.70]	1.80 [1.30;2.70]	1.80 [1.20;2.80]	2.30 [1.40;3.58]	<0.001
PCO2	40.0 [34.0;46.0]	39.0 [35.0;43.0]	40.0 [34.0;46.9]	40.0 [34.0;46.0]	40.0 [34.2;47.0]	0.253
PO2	126 [73.0;220]	154 [87.0;222]	120 [73.2;198]	127 [73.0;224]	111 [65.0;221]	0.083
INR	1.30 [1.10;1.50]	1.20 [1.10;1.40]	1.20 [1.10;1.40]	1.20 [1.10;1.50]	1.32 [1.20;1.60]	<0.001
PT	13.8 [12.5;16.6]	12.9 [12.0;15.1]	13.4 [12.3;15.2]	13.7 [12.4;16.3]	14.8 [13.0;17.8]	<0.001
PTT	30.1 [26.4;36.6]	29.2 [26.0;33.8]	28.0 [25.3;32.5]	30.2 [26.7;36.8]	32.1 [28.0;38.9]	<0.001
ALT	34.0 [19.0;72.0]	31.0 [18.5;62.1]	33.0 [20.0;73.5]	33.0 [18.0;67.0]	37.0 [20.0;81.0]	0.270
AST	49.0 [28.0;116]	45.0 [26.5;80.5]	46.5 [27.2;94.8]	45.0 [27.0;110]	61.0 [31.0;142]	0.004
TB	0.60 [0.40;1.10]	0.60 [0.50;1.00]	0.60 [0.40;1.10]	0.60 [0.40;1.00]	0.60 [0.40;1.30]	0.496
CRE	1.00 [0.80;1.50]	0.90 [0.70;1.20]	0.90 [0.70;1.20]	1.00 [0.80;1.50]	1.20 [0.80;1.70]	<0.001
MV:						0.223
No	244 (19.9%)	30 (19.9%)	47 (24.2%)	102 (20.5%)	65 (17.0%)	
Yes	980 (80.1%)	121 (80.1%)	147 (75.8%)	395 (79.5%)	317 (83.0%)	
VP:						0.223
No	349 (28.5%)	53 (35.1%)	53 (27.3%)	143 (28.8%)	100 (26.2%)	
Yes	875 (71.5%)	98 (64.9%)	141 (72.7%)	354 (71.2%)	282 (73.8%)	
SA:						0.593
No	97 (7.92%)	10 (6.62%)	16 (8.25%)	45 (9.05%)	26 (6.81%)	
Yes	1,127 (92.1%)	141 (93.4%)	178 (91.8%)	452 (90.9%)	356 (93.2%)	
CRRT:						0.558
No	1,102 (90.0%)	132 (87.4%)	173 (89.2%)	448 (90.1%)	349 (91.4%)	
Yes	122 (9.97%)	19 (12.6%)	21 (10.8%)	49 (9.86%)	33 (8.64%)	
URE	19.0 [13.0;31.0]	15.0 [11.0;23.0]	16.0 [12.0;23.0]	19.0 [14.0;30.0]	23.0 [15.0;38.8]	<0.001
Hosp time	16.8 [10.3;27.2]	18.4 [10.8;28.0]	15.2 [10.6;23.5]	16.8 [9.93;27.7]	17.6 [10.5;27.5]	0.250
Hosp dead	346 (28.3%)	20 (13.2%)	39 (20.1%)	152 (30.6%)	135 (35.3%)	<0.001
ICU time	9.09 [4.86;16.0]	10.1 [4.89;15.6]	8.64 [5.69;14.6]	8.81 [4.86;16.0]	9.34 [4.58;16.7]	0.967
ICU dead	292 (23.9%)	19 (12.6%)	24 (12.4%)	130 (26.2%)	119 (31.2%)	<0.001

### Survival analysis

**Figure 1:**
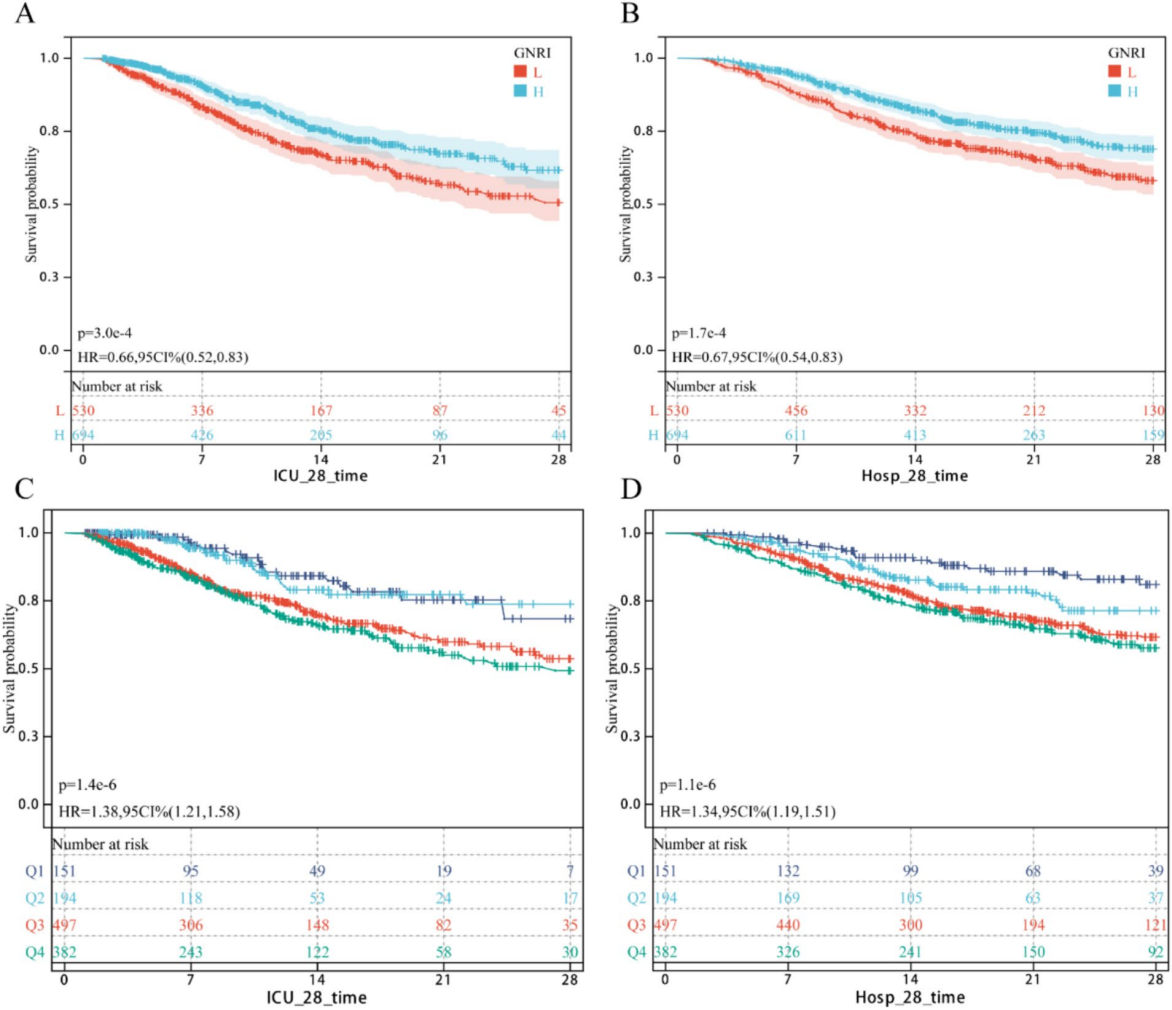
KM survival curv. **(A,B)** Continuous GNRI; **(C,D)** Classification GNRI.

As shown in Figure 1, Kaplan–Meier survival curves confirmed a significant inverse correlation between higher GNRI scores and reduced mortality. Lower GNRI groups exhibited significantly higher 28-day ICU mortality (HR = 0.66, 95% CI: 0.55–0.83) and 28-day hospital all-cause mortality (HR = 0.67, 0.54–0.83) compared to higher GNRI groups. Similarly, patients with nutritional risk had markedly elevated mortality versus the no-risk group: 28-day ICU mortality (HR = 1.38, 1.21–1.58) and 28-day all-cause mortality (HR = 1.34, 1.19–1.51).

### Multivariate Cox regression analysis of the association between GNRI and short-term mortality in cerebral critical care patients

**Figure 2:**
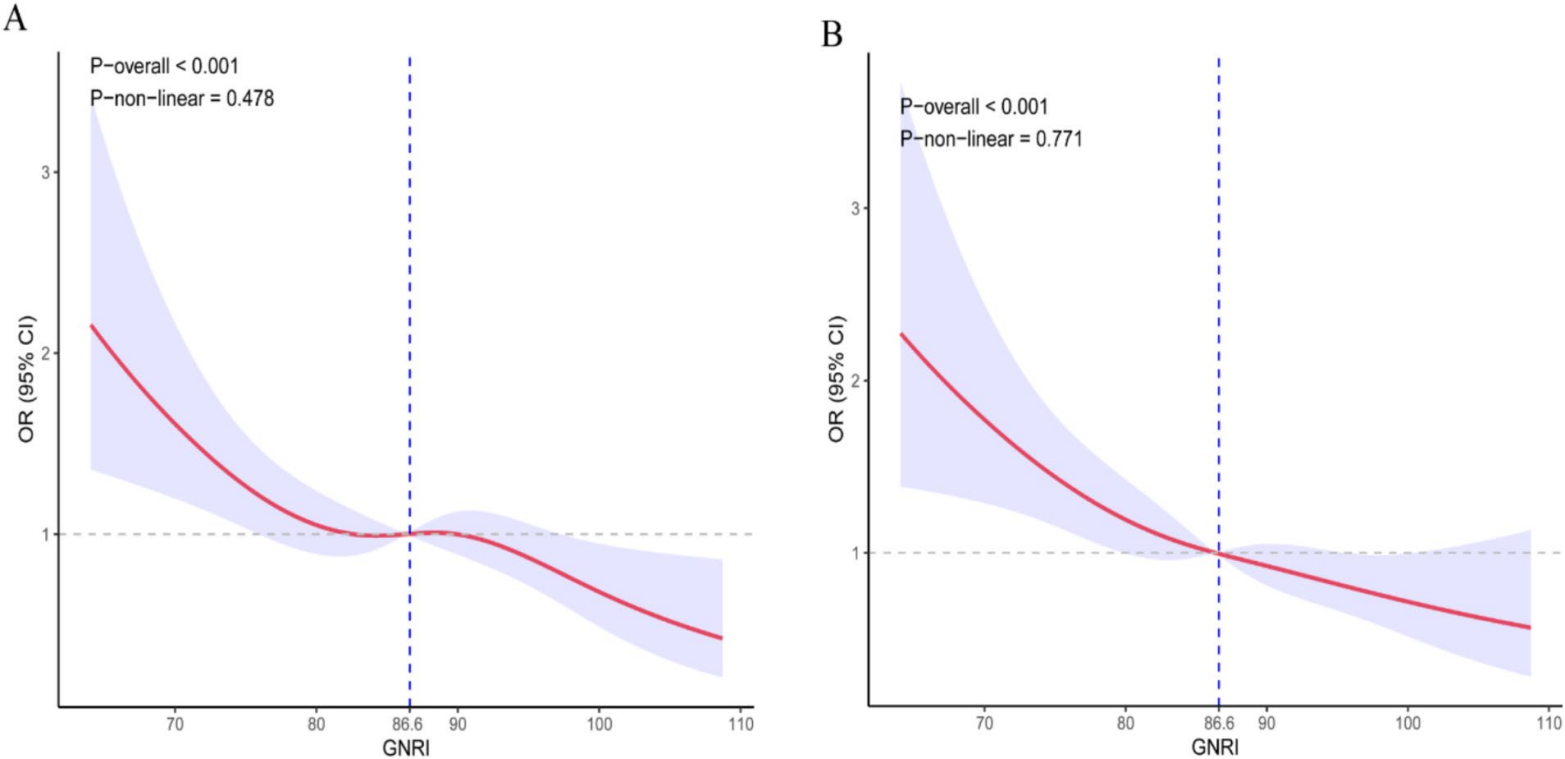
Dose–response curve of GNRI and short-term mortality rate. **(A)** ICU death; **(B)** Hospitalized death.

As shown in Table 2, the multivariable Cox proportional hazards model adjusted for all potential covariates revealed a significant inverse association between higher GNRI scores and 28-day ICU mortality (HR = 0.97, 95% CI: 0.96–0.99). Compared to the no-risk group, patients with moderate and high nutritional risk exhibited increased mortality (HR = 1.66, 1.01–2.74, and HR = 1.79, 1.08–2.97, respectively). Similarly, for 28-day hospital all-cause mortality (Table 3), higher GNRI scores were inversely associated with reduced risk (HR = 0.97, 0.96–0.99). Consistently, moderate- and high-risk groups showed elevated mortality versus the no-risk group (HR = 2.03, 1.26–3.28 and HR = 2.11, 1.30–3.42, respectively). Trend analysis further confirmed a dose–response relationship between escalating nutritional risk and both 28-day ICU/hospital mortality (*p* < 0.05). RCS Shows Inverse Dose Relationship Between GNRI and Short-Term Mortality in Cerebral Critical Care Patients (nonlinearity *p* = 0.478 for ICU mortality; *p* = 0.771 for hospital mortality; Figures 2A,B).

**Table 2:** The relationship between GNRI and 28 day ICU mortality rate.

	Model 1			Model 2			Model 3		
Characteristic	HR^1^	95% CI^1^	*p*-value	HR^1^	95% CI^1^	*p*-value	HR^1^	95% CI^1^	*p*-value
GNRI	0.96	0.95, 0.97	<0.001	0.96	0.95, 0.98	<0.001	0.97	0.96, 0.99	<0.001
GNRI group									
No risk	Ref	Ref		Ref	Ref		Ref	Ref	
Low risk	1.01	0.56, 1.85	0.9	0.91	0.50, 1.67	0.8	0.92	0.50, 1.69	0.8
Moderate risk	2.07	1.28, 3.35	0.003	1.78	1.10, 2.90	0.019	1.66	1.01, 2.74	0.046
High risk	2.42	1.49, 3.93	<0.001	2.15	1.32, 3.49	0.002	1.79	1.08, 2.97	0.023

^1^HR, Hazard Ratio; CI, Confidence Interval.

**Table 3:** The relationship between GNRI and 28 day hospital mortality rate.

	Model 1			Model 2			Model 3		
Characteristic	HR^1^	95% CI^1^	*p*-value	HR^1^	95% CI^1^	*p*-value	HR^1^	95% CI^1^	*p*-value
GNRI	0.96	0.95, 0.98	<0.001	0.97	0.96, 0.98	<0.001	0.97	0.96, 0.99	<0.001
GNRI group									
No risk	Ref	Ref		Ref	Ref		Ref	Ref	
Low risk	1.69	0.98, 2.89	0.057	1.54	0.90, 2.64	0.12	1.57	0.91, 2.71	0.10
Moderate risk	2.44	1.53, 3.89	<0.001	2.10	1.31, 3.36	0.002	2.03	1.26, 3.28	0.004
High risk	2.80	1.75, 4.48	<0.001	2.42	1.51, 3.87	<0.001	2.11	1.30, 3.42	0.002

^1^HR, Hazard Ratio; CI, Confidence Interval.

### Subgroup analysis

To assess the association between GNRI and 28-day ICU/hospital mortality across demographic and clinical subgroups, we conducted stratified analyses. In the fully adjusted model, results (Supplementary Figures S1, S2) demonstrated that the GNRI-mortality relationship remained consistent regardless of sex, ethnicity, obesity, hypertension, AKI, or CKD. However, this relationship varied by age, diabetes status, and COPD: GNRI showed no statistically significant association with mortality in younger patients (<65 years), those with diabetes, or COPD. Interaction tests confirmed no significant effect modification between GNRI and these subgroups (all *p* > 0.05).

### Construction of the nomogram

**Figure 3:**
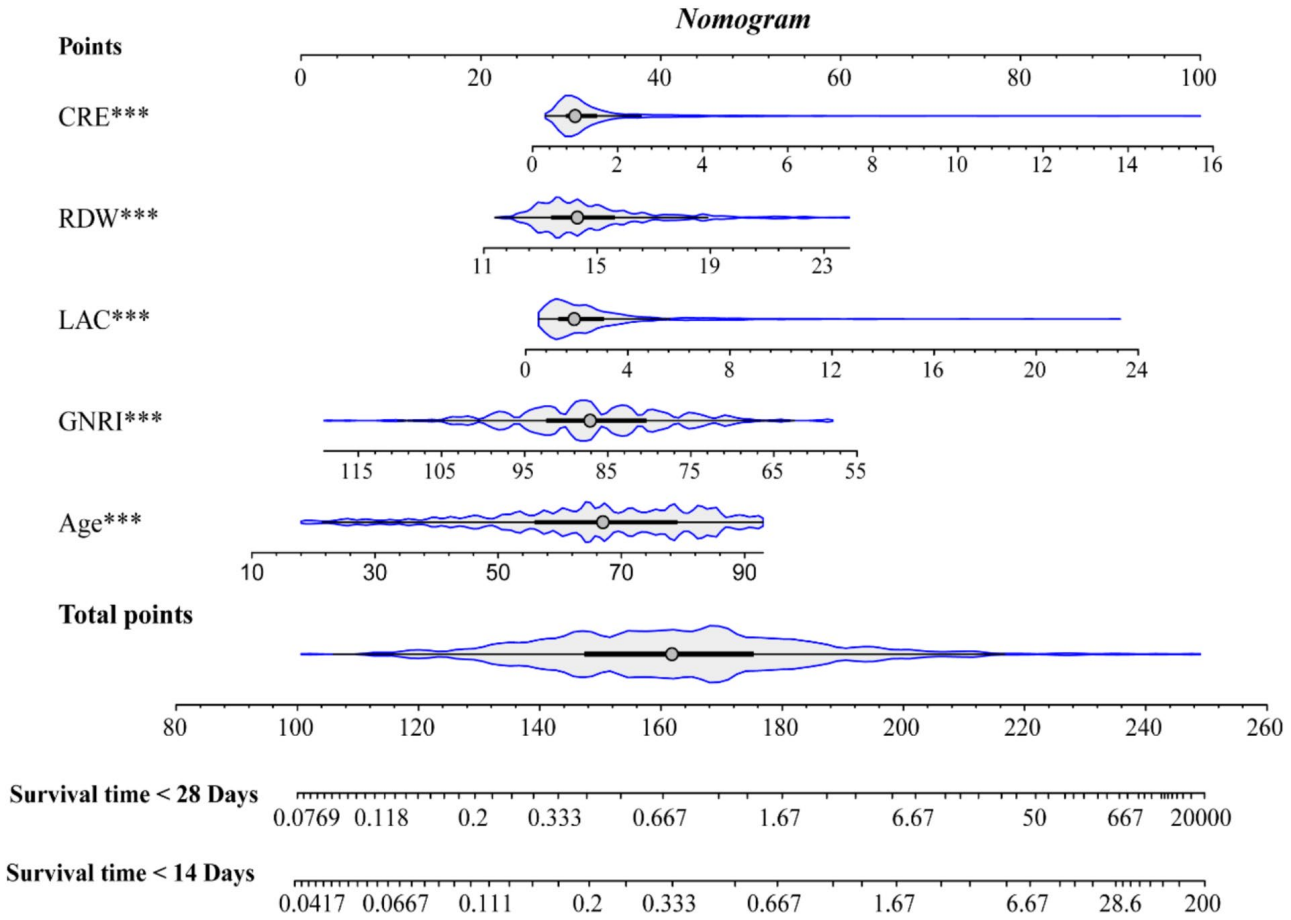
Nomogram.

**Figure 4:**
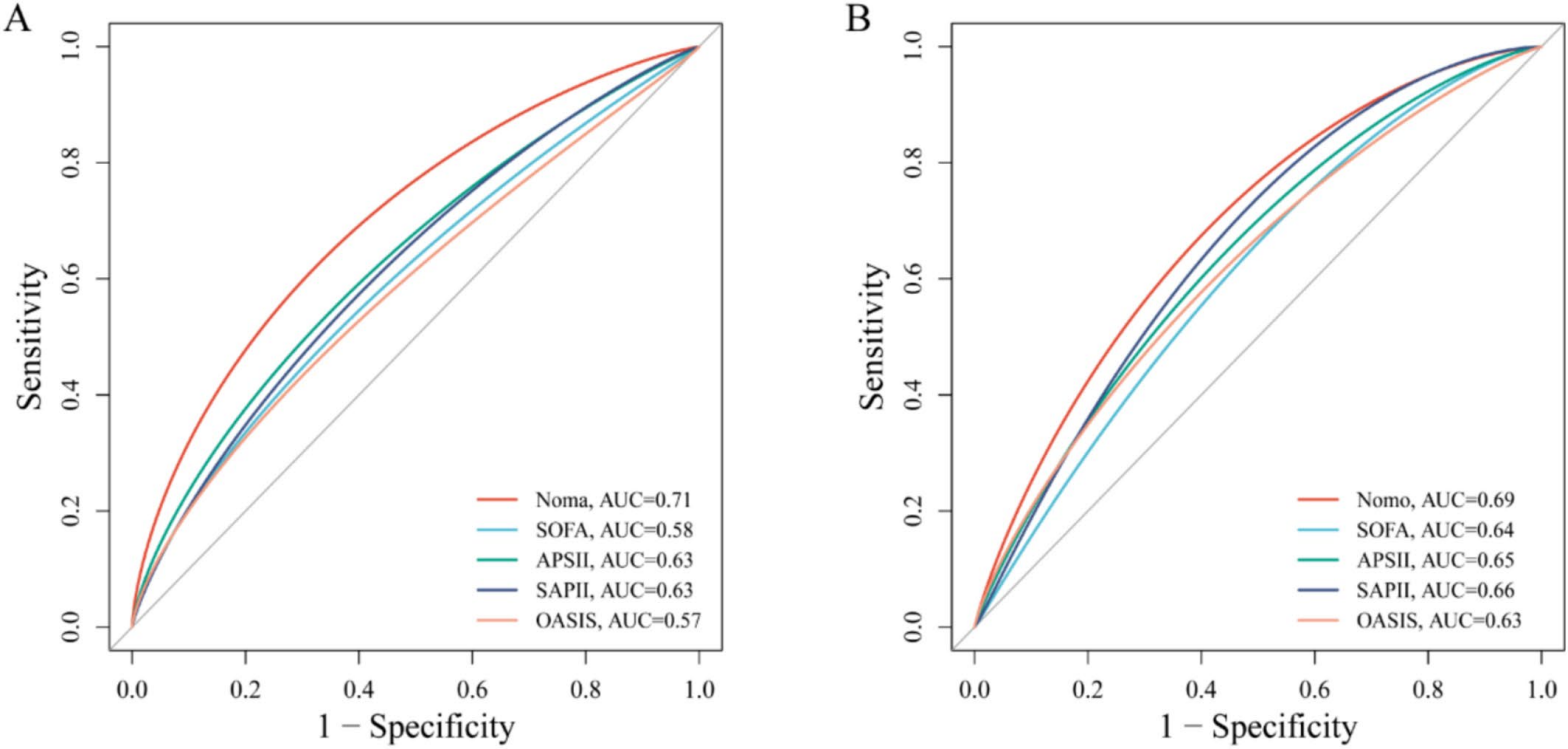
ROC curve comparing the effectiveness of column chart and traditional severity score. **(A)** Training set; **(B)** Test set.

In the training cohort, 49 candidate variables were analyzed using LASSO regression (Supplementary Figures S3A,B) and multivariable Cox regression. Five independent predictors—GNRI, age, red cell distribution width (RDW), lactate (LAC), and creatinine (CRE)—were selected to construct a risk prediction model for 28-day ICU mortality in critically ill neurological patients (Figure 3). The nomogram demonstrated superior sensitivity and specificity in predicting 28-day ICU mortality compared to traditional severity scores (SOFA, APS III, SAPS II, OASIS), with AUROC values of 0.72 (training cohort; Figure 4A) and 0.69 (validation cohort; Figure 4B).

### External queue verification

**Figure 5:**
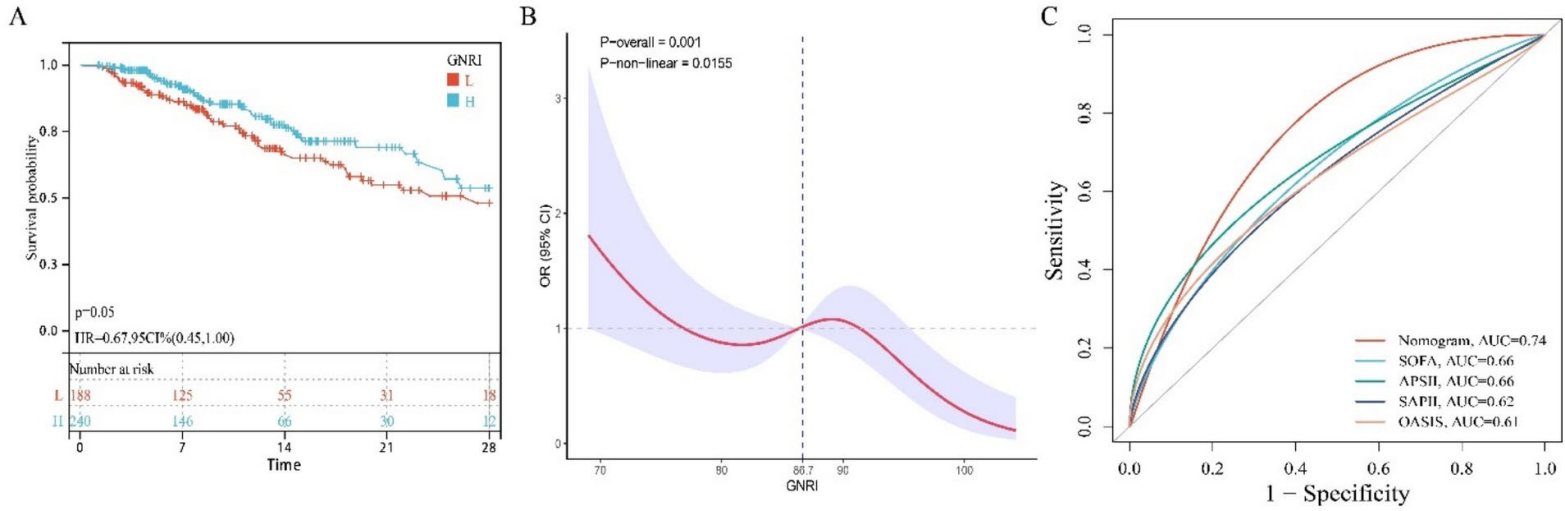
The relationship between GNRI and mortality rate in external queues. **(A)** KM survival curve; **(B)** RCS curve; **(C)** ROC curve.

In an external cohort of 428 neurocritical care patients (28-day mortality rate = 23.60%). Multivariable Cox proportional hazards regression confirmed that higher GNRI scores were significantly associated with reduced 28-day mortality risk (HR = 0.95; 95% CI: 0.93–0.98) (Table 4). Similarly, patients with high nutritional risk demonstrated significantly increased mortality compared to those without nutritional risk (HR = 3.75; 95% CI: 1.21–11.7) (Table 4). Kaplan–Meier curves further demonstrated significantly lower 28-day ICU mortality in patients with GNRI scores above the cohort median (log-rank *p* < 0.05; HR = 0.67, 95% CI: 0.45–1.00; Figure 5A). Restricted cubic spline analysis revealed a significant inverse dose–response relationship between GNRI and mortality (*p* = 0.001; Figure 5B). The prognostic nomogram demonstrated robust external validation performance (Figure 5C), with AUC of 0.74 and good calibration between predicted and observed mortality probabilities.

**Table 4:** The relationship between GNRI and 28 day mortality rate of external cohort patients.

	Model 1			Model 2			Model 3		
Characteristic	HR^1^	95% CI^1^	*p*-value	HR^1^	95% CI^1^	*p*-value	HR^1^	95% CI^1^	*p*-value
GNRI	0.95	0.93, 0.97	<0.001	0.96	0.93, 0.98	<0.001	0.95	0.93, 0.98	<0.001
GNRI group									
No risk	Ref	Ref		Ref	Ref		Ref	Ref	
Low risk	0.69	0.20, 2.46	0.6	0.63	0.18, 2.27	0.5	1.15	0.27, 4.92	0.9
Moderate risk	2.58	1.10, 6.06	0.029	2.00	0.84, 4.76	0.12	3.16	1.03, 9.65	0.043
High risk	3.33	1.42, 7.80	0.006	2.47	1.04, 5.86	0.040	3.75	1.21, 11.7	0.022

^1^HR, Hazard Ratio; CI, Confidence Interval.

## Discussion

Cerebral injury remains a leading global cause of long-term disability and mortality, posing significant challenges to public health systems (1–3). Previous studies have emphasized the need to identify reliable and easily measurable biomarkers to improve clinical outcomes in CIPCI (18–21). This study is the first to establish a clear association between the GNRI and mortality in CIPCI. Our findings demonstrate that lower GNRI levels are an independent predictor of 28-day ICU and hospital all-cause mortality, even after adjusting for potential confounders. A linear dose–response relationship was observed between GNRI and mortality, with this association remaining consistent across diverse demographic and clinical subgroups. Notably, when analyzed categorically, patients with moderate or high nutritional risk exhibited significantly higher mortality compared to the no-risk group. Finally, we provide a simple and effective tool for risk stratification, allowing clinicians to identify at-risk individuals and provide timely intervention.

Malnutrition, defined as an imbalance between energy intake and physiological demands (22), is strongly linked to cerebral injury (4, 5, 23). While the underlying mechanisms are multifactorial, inflammation has been identified as a central mediator in this pathological interplay (24–27). First, emerging evidence suggests that inflammatory responses exacerbate secondary brain injury after hemorrhagic events and correlate strongly with poor clinical outcomes (26–28). Malnutrition and inflammation exhibit bidirectional interactions (24, 25). For instance, hypoalbuminemia—a hallmark of malnutrition—is associated with systemic hyperinflammation (29, 30). Conversely, excessive inflammation suppresses albumin synthesis, perpetuating malnutrition and creating a self-reinforcing cycle of adverse outcomes (24, 25, 29–31). Additionally, malnutrition may trigger oxidative stress, impaired insulin signaling, lipid peroxidation, immune dysregulation, and accelerated aging (30, 32, 33), all of which amplify tissue damage and accelerate cerebral injury progression (34).

The GNRI is a simple yet effective tool for assessing malnutrition risk and predicting clinical outcomes in older adults (10). Prior studies have validated its prognostic utility in ICU populations, linking low GNRI scores to increased mortality and adverse outcomes in critical conditions such as sepsis, trauma, and acute kidney injury (11–14). Consistent with these findings, our study highlights a significant association between GNRI-defined nutritional risk and poor prognosis in CIPCI. Notably, our analysis extends prior work by including a broader, more heterogeneous cohort of cerebral injury critical illnesses. For example, Serrato et al. associated low GNRI with prolonged hospitalization, higher comorbidity burden, adverse events, and non-routine discharge in elderly patients undergoing subdural hematoma evacuation (35). Similarly, other studies have linked GNRI to ischemic stroke incidence and poor post-stroke outcomes (8, 9, 36). However, these investigations focused on specific cerebral injury subtypes, relied on smaller single-center cohorts, and did not explore integrating GNRI with routine clinical data to develop predictive models.

Furthermore, our subgroup analyses demonstrated that the association between GNRI and adverse outcomes in CIPCI remained consistent across sex, ethnicity, obesity, hypertension, AKI, and CKD. In contrast, no statistically significant association was observed in younger patients or those with diabetes or COPD. This lack of association may reflect limited sample size in these subgroups or disease-specific metabolic alterations, underscoring the need for further validation in larger, more diverse ICU populations.

Notably, we developed a predictive model to estimate 28-day ICU mortality risk in critically ill neurological patients. This model, visualized as a clinician-friendly nomogram, enables mortality risk assessment using routinely collected clinical data and demonstrates superior sensitivity and specificity compared to traditional severity scores. The model achieved an Area under the ROC Curve (AUC) of 0.72 (training cohort) and 0.69 (validation cohort). Healthcare providers can stratify mortality risk for neurological ICU admissions within minutes. This tool provides actionable insights to guide time-sensitive clinical decisions through individualized risk stratification.

While this study demonstrates GNRI’s independent prognostic value for short-term outcomes in neurocritical patients and establishes a clinically applicable risk prediction model, several limitations warrant acknowledgment. First, the retrospective observational design precludes causal inference between GNRI and mortality. Second, despite rigorous adjustment for known confounders, the absence of neuroimaging data (e.g., hematoma location/volume, cerebral edema) and standardized severity scores makes it exceptionally challenging to disentangle the independent effects of “nutritional status” from those of “initial brain injury severity.” Unmeasured initial neurological injury severity constitutes a potent confounder that likely significantly influenced the observed associations, thereby weakening confidence in conclusions regarding nutritional status’ independent role. Furthermore, nutritional assessment limited to the first 24 h likely reflects acute illness/injury severity and early stress response at ICU admission rather than sustained nutritional status. Consequently, these findings cannot represent nutritional exposures during the entire ICU stay—particularly during the rehabilitation phase critical for long-term functional recovery. Finally, while externally validated in a real-world cohort, generalizability remains constrained by the single-center design and limited sample size. In summary, future studies with larger cohorts and enhanced designs are needed to clarify GNRI’s relationship with short-term outcomes in neurocritical patients.

## Conclusion

This study revealed a significant negative correlation between the GNRI and short-term mortality in critically ill patients with cerebral injury. Furthermore, we developed a clinically valuable risk stratification tool based on GNRI and routinely collected clinical data, enabling clinicians to identify high-risk individuals and prioritize timely interventions.

## Data Availability

The original contributions presented in the study are included in the article/Supplementary material, further inquiries can be directed to the corresponding author/s.
