# Supplementary figures and images for: The relationship between elderly nutritional risk index and short-term all-cause mortality in critically ill patients with cerebral injury: a retrospective cohort study from two cohorts
Source: Front Nutr. 2025 Jul 24;12:1620364. doi: 10.3389/fnut.2025.1620364 (PMC12328167; doi:10.3389/fnut.2025.1620364)

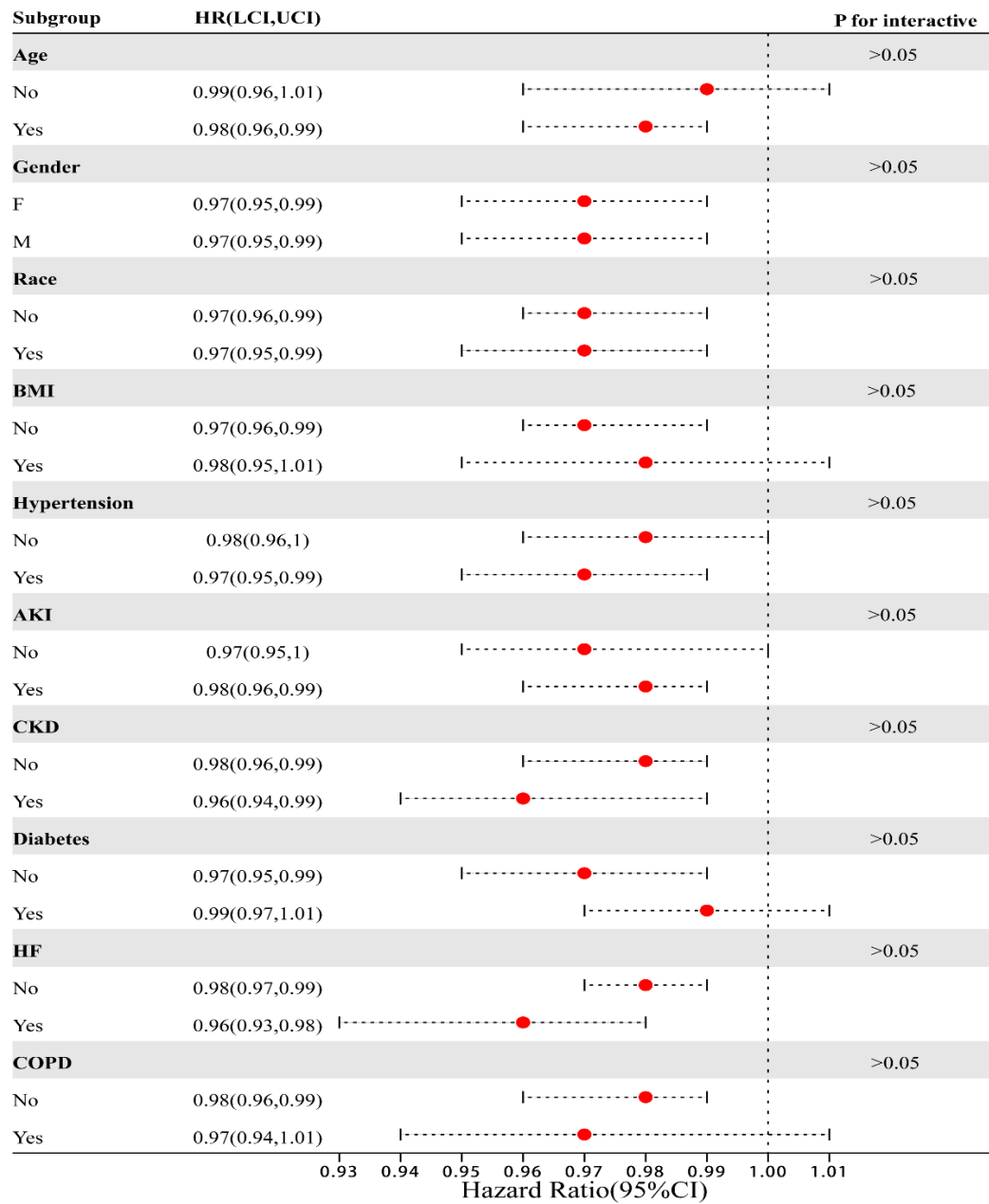

Fig S1: Subgroup analysis of GNRI and 28day ICU mortality rate

Supplement: Supplementary file 2 [file Image_1.pdf]

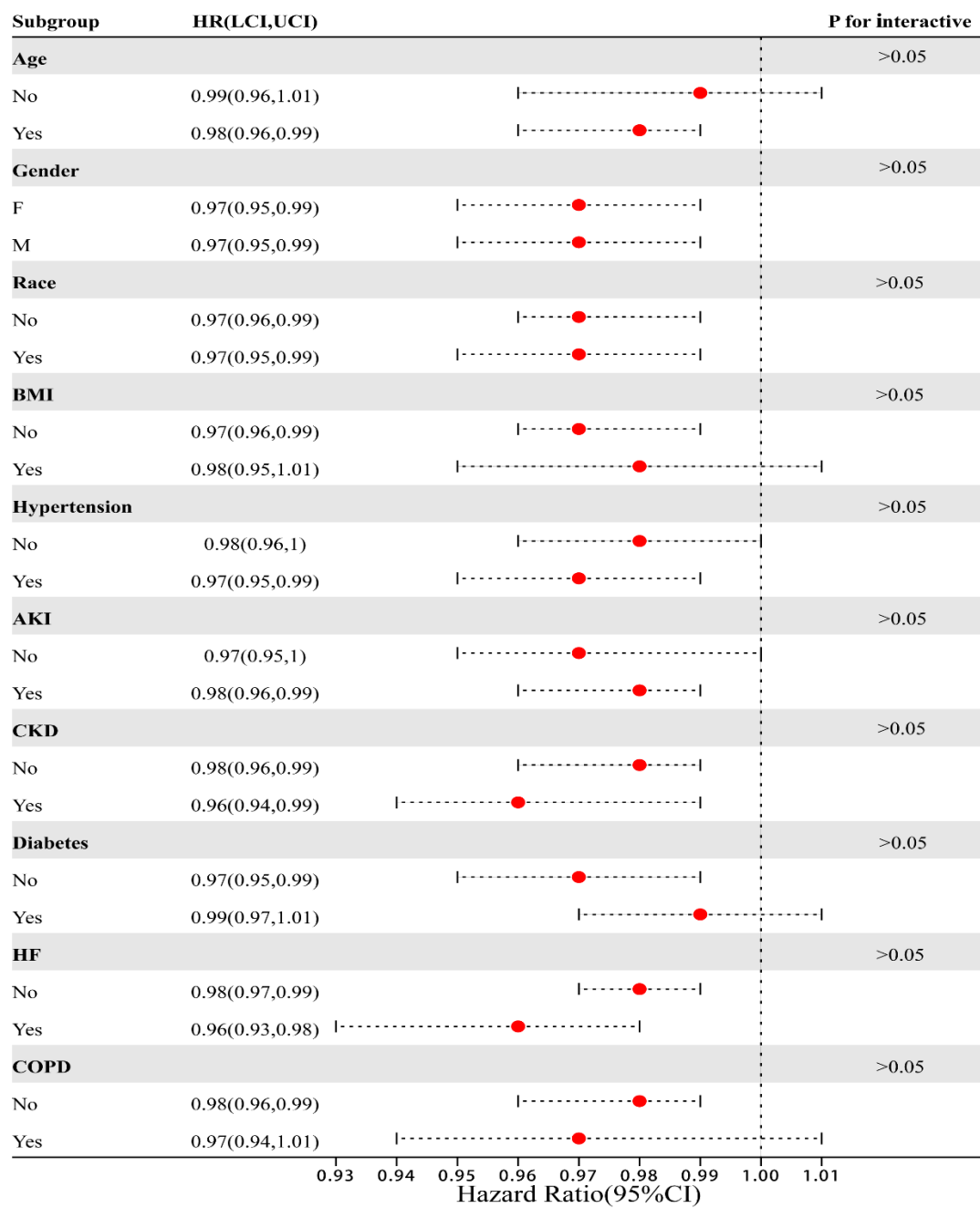

Fig S2: Subgroup analysis of GNRI and 28day hospital mortality rate

Supplement: Supplementary file 3 [file Image_2.pdf]

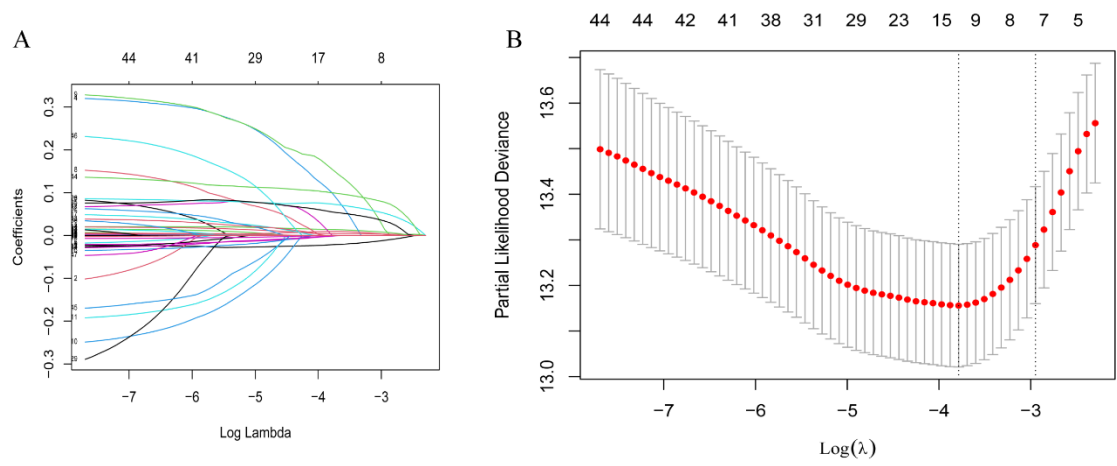

Fig S3: The convergence process of Lasso regression on candidate variables

Supplement: Supplementary file 4 [file Image_3.pdf]
