# Supplementary material for: The relationship between elderly nutritional risk index and short-term all-cause mortality in critically ill patients with cerebral injury: a retrospective cohort study from two cohorts
Source: Front Nutr. 2025 Jul 24;12:1620364. doi: 10.3389/fnut.2025.1620364 (PMC12328167; doi:10.3389/fnut.2025.1620364)
Supplement: Supplementary file 5 [file Table_1.docx]

Table S1: Summary descriptives table by groups of 28day in-ICU mortality rate

|  | **ALL** | **Survivor** | **No-survivor** | **P-value** |
| --- | --- | --- | --- | --- |
|  | **N=1224** | **N=932** | **N=292** |  |
| GNRI | 86.4[80.4;92.3] | 87.9[81.9;93.8] | 84.9[77.6;89.3] | <0.001 |
| GNRI group: |  |  |  | <0.001 |
| Nor isk | 151(12.3%) | 132(14.2%) | 19(6.51%) |  |
| Low risk | 194(15.8%) | 170(18.2%) | 24(8.22%) |  |
| Moderate risk | 497(40.6%) | 367(39.4%) | 130(44.5%) |  |
| High risk | 382(31.2%) | 263(28.2%) | 119(40.8%) |  |
| Age | 67.0[55.0;79.0] | 66.0[53.8;78.0] | 71.5[60.0;82.0] | <0.001 |
| Gender: |  |  |  | 0.032 |
| F | 461(37.7%) | 335(35.9%) | 126(43.2%) |  |
| M | 763(62.3%) | 597(64.1%) | 166(56.8%) |  |
| Race: |  |  |  | 0.210 |
| No | 525(42.9%) | 390(41.8%) | 135(46.2%) |  |
| Yes | 699(57.1%) | 542(58.2%) | 157(53.8%) |  |
| Weight | 80.0[68.1;94.9] | 80.5[69.0;96.0] | 77.8[67.2;92.0] | 0.022 |
| Height | 170[163;178] | 173[163;178] | 168[163;178] | 0.012 |
| BMI | 27.3[24.0;31.9] | 27.6[24.1;31.9] | 26.8[23.6;31.8] | 0.151 |
| Hypertension: |  |  |  | 0.172 |
| No | 668(54.6%) | 498(53.4%) | 170(58.2%) |  |
| Yes | 556(45.4%) | 434(46.6%) | 122(41.8%) |  |
| AKI: |  |  |  | <0.001 |
| No | 670(54.7%) | 547(58.7%) | 123(42.1%) |  |
| Yes | 554(45.3%) | 385(41.3%) | 169(57.9%) |  |
| CKD: |  |  |  | 0.001 |
| No | 1004(82.0%) | 784(84.1%) | 220(75.3%) |  |
| Yes | 220(18.0%) | 148(15.9%) | 72(24.7%) |  |
| Diabetes: |  |  |  | 0.885 |
| No | 853(69.7%) | 651(69.8%) | 202(69.2%) |  |
| Yes | 371(30.3%) | 281(30.2%) | 90(30.8%) |  |
| HF: |  |  |  | 0.017 |
| No | 882(72.1%) | 688(73.8%) | 194(66.4%) |  |
| Yes | 342(27.9%) | 244(26.2%) | 98(33.6%) |  |
| COPD: |  |  |  | 0.026 |
| No | 1022(83.5%) | 791(84.9%) | 231(79.1%) |  |
| Yes | 202(16.5%) | 141(15.1%) | 61(20.9%) |  |
| SOFA | 6.00[3.00;9.00] | 5.00[3.00;8.00] | 7.00[4.00;10.0] | <0.001 |
| APSII | 49.0[36.0;66.0] | 47.0[34.0;61.0] | 58.0[43.8;78.2] | <0.001 |
| SAPII | 40.0[31.0;51.0] | 38.5[29.0;49.0] | 46.0[37.0;58.0] | <0.001 |
| OASIS | 36.0[31.0;42.0] | 36.0[30.0;42.0] | 39.0[32.0;45.0] | <0.001 |
| GCS | 15.0[11.0;15.0] | 15.0[11.0;15.0] | 15.0[12.0;15.0] | 0.091 |
| HR | 87.0[75.0;101] | 87.0[75.0;101] | 87.0[75.0;102] | 0.646 |
| RR | 19.0[16.0;22.0] | 18.0[15.0;22.0] | 19.5[16.0;23.2] | 0.022 |
| NBPS | 124[105;143] | 125[106;143] | 120[100;141] | 0.002 |
| NBPD | 68.0[57.0;81.0] | 69.0[57.0;82.0] | 66.0[54.8;79.0] | 0.026 |
| NBPM | 82.0[70.0;95.0] | 83.0[71.0;95.0] | 80.0[66.0;94.0] | 0.005 |
| HCT | 33.5[28.7;38.2] | 33.8[28.9;38.6] | 32.7[28.0;37.3] | 0.050 |
| Hb | 11.1[9.40;12.7] | 11.2[9.50;12.9] | 10.7[9.10;12.2] | 0.003 |
| PLT | 192[137;255] | 192[138;252] | 188[131;265] | 0.915 |
| RDW | 14.3[13.4;15.6] | 14.2[13.3;15.3] | 14.9[13.8;16.4] | <0.001 |
| RBC | 3.67[3.14;4.22] | 3.73[3.17;4.27] | 3.58[3.01;4.09] | 0.015 |
| WBC | 12.3[8.88;16.2] | 12.1[8.70;15.7] | 13.1[9.38;18.4] | 0.002 |
| ALB | 3.10[2.70;3.50] | 3.10[2.70;3.50] | 2.90[2.50;3.20] | <0.001 |
| AG | 15.0[12.0;17.0] | 14.0[12.0;17.0] | 16.0[13.0;19.0] | <0.001 |
| Glu | 142[115;183] | 138[114;179] | 155[117;197] | 0.007 |
| K | 4.00[3.70;4.50] | 4.00[3.70;4.50] | 4.10[3.80;4.70] | 0.004 |
| Na | 139[137;142] | 139[137;142] | 139[136;143] | 0.867 |
| CL | 105[101;109] | 105[102;109] | 105[100;109] | 0.062 |
| LAC | 1.90[1.30;3.00] | 1.90[1.30;2.90] | 2.29[1.30;3.60] | 0.001 |
| PCO2 | 40.0[34.0;46.0] | 40.0[35.0;46.0] | 40.0[34.0;48.2] | 0.433 |
| PO2 | 126[73.0;220] | 131[78.0;224] | 108[62.0;203] | 0.005 |
| INR | 1.30[1.10;1.50] | 1.20[1.10;1.50] | 1.32[1.20;1.70] | <0.001 |
| PT | 13.8[12.5;16.6] | 13.7[12.4;16.1] | 14.9[12.8;18.6] | <0.001 |
| PTT | 30.1[26.4;36.6] | 29.6[26.3;35.5] | 32.0[27.9;40.0] | <0.001 |
| ALT | 34.0[19.0;72.0] | 33.0[19.0;70.0] | 36.0[20.0;91.0] | 0.203 |
| AST | 49.0[28.0;116] | 47.0[27.0;105] | 57.0[30.9;164] | 0.003 |
| TB | 0.60[0.40;1.10] | 0.60[0.40;1.10] | 0.60[0.40;1.30] | 0.305 |
| CRE | 1.00[0.80;1.50] | 1.00[0.80;1.40] | 1.20[0.80;1.90] | <0.001 |
| URE | 19.0[13.0;31.0] | 18.0[13.0;28.0] | 23.0[15.0;38.0] | <0.001 |
| MV: |  |  |  | 0.164 |
| No | 244(19.9%) | 177(19.0%) | 67(22.9%) |  |
| Yes | 980(80.1%) | 755(81.0%) | 225(77.1%) |  |
| VP: |  |  |  | 0.971 |
| No | 349(28.5%) | 265(28.4%) | 84(28.8%) |  |
| Yes | 875(71.5%) | 667(71.6%) | 208(71.2%) |  |
| SA: |  |  |  | 0.020 |
| No | 97(7.92%) | 64(6.87%) | 33(11.3%) |  |
| Yes | 1127(92.1%) | 868(93.1%) | 259(88.7%) |  |
| CRRT: |  |  |  | 0.303 |
| No | 1102(90.0%) | 834(89.5%) | 268(91.8%) |  |
| Yes | 122(9.97%) | 98(10.5%) | 24(8.22%) |  |
