# Supplementary material for: The relationship between elderly nutritional risk index and short-term all-cause mortality in critically ill patients with cerebral injury: a retrospective cohort study from two cohorts
Source: Front Nutr. 2025 Jul 24;12:1620364. doi: 10.3389/fnut.2025.1620364 (PMC12328167; doi:10.3389/fnut.2025.1620364)
Supplement: Supplementary file 6 [file Table_2.docx]

TableS2: Summary descriptives table by groups of 28day in-hospital all-cause mortality rate

|  | **ALL** | **Survivor** | **No-survivor** | **P-value** |
| --- | --- | --- | --- | --- |
|  | **N=1224** | **N=878** | **N=346** |  |
| GNRI | 86.4[80.4;92.3] | 87.9[81.9;93.8] | 84.9[78.9;89.3] | <0.001 |
| GNRI group: |  |  |  | <0.001 |
| No risk | 151(12.3%) | 131(14.9%) | 20(5.78%) |  |
| Low risk | 194(15.8%) | 155(17.7%) | 39(11.3%) |  |
| Moderate risk | 497(40.6%) | 345(39.3%) | 152(43.9%) |  |
| High risk | 382(31.2%) | 247(28.1%) | 135(39.0%) |  |
| Age | 67.0[55.0;79.0] | 65.0[53.0;76.0] | 73.0[62.0;83.0] | <0.001 |
| Gender: |  |  |  | 0.034 |
| F | 461(37.7%) | 314(35.8%) | 147(42.5%) |  |
| M | 763(62.3%) | 564(64.2%) | 199(57.5%) |  |
| Race: |  |  |  | 0.014 |
| No | 525(42.9%) | 357(40.7%) | 168(48.6%) |  |
| Yes | 699(57.1%) | 521(59.3%) | 178(51.4%) |  |
| Weight | 80.0[68.1;94.9] | 80.8[69.0;96.0] | 78.2[67.5;92.0] | 0.010 |
| Height | 170[163;178] | 173[163;178] | 169[163;178] | 0.006 |
| BMI | 27.3[24.0;31.9] | 27.6[24.1;31.9] | 26.9[23.8;31.8] | 0.134 |
| HYT |  |  |  | 1.000 |
| No | 668(54.6%) | 479(54.6%) | 189(54.6%) |  |
| Yes | 556(45.4%) | 399(45.4%) | 157(45.4%) |  |
| AKI: |  |  |  | <0.001 |
| No | 670(54.7%) | 513(58.4%) | 157(45.4%) |  |
| Yes | 554(45.3%) | 365(41.6%) | 189(54.6%) |  |
| CKD: |  |  |  | 0.002 |
| No | 1004(82.0%) | 739(84.2%) | 265(76.6%) |  |
| Yes | 220(18.0%) | 139(15.8%) | 81(23.4%) |  |
| Diabetes: |  |  |  | 0.360 |
| No | 853(69.7%) | 619(70.5%) | 234(67.6%) |  |
| Yes | 371(30.3%) | 259(29.5%) | 112(32.4%) |  |
| HF: |  |  |  | 0.036 |
| No | 882(72.1%) | 648(73.8%) | 234(67.6%) |  |
| Yes | 342(27.9%) | 230(26.2%) | 112(32.4%) |  |
| COPD: |  |  |  | 0.022 |
| No | 1022(83.5%) | 747(85.1%) | 275(79.5%) |  |
| Yes | 202(16.5%) | 131(14.9%) | 71(20.5%) |  |
| SOFA | 6.00[3.00;9.00] | 5.00[3.00;8.00] | 7.00[4.00;10.0] | <0.001 |
| APSII | 49.0[36.0;66.0] | 47.0[34.0;61.0] | 57.0[43.0;77.8] | <0.001 |
| SAPII | 40.0[31.0;51.0] | 38.0[29.0;49.0] | 47.0[37.0;57.0] | <0.001 |
| OASIS | 36.0[31.0;42.0] | 35.0[30.0;41.0] | 39.0[33.0;46.0] | <0.001 |
| GCS | 15.0[11.0;15.0] | 15.0[11.0;15.0] | 15.0[11.0;15.0] | 0.494 |
| HR | 87.0[75.0;101] | 87.0[75.0;101] | 87.0[75.0;101] | 0.952 |
| RR | 19.0[16.0;22.0] | 18.0[15.0;22.0] | 19.0[16.0;22.0] | 0.195 |
| NBPS | 124[105;143] | 125[106;144] | 120[100;139] | 0.002 |
| NBPD | 68.0[57.0;81.0] | 69.0[58.0;82.0] | 66.0[54.2;79.0] | 0.015 |
| NBPM | 82.0[70.0;95.0] | 83.0[71.0;95.8] | 80.0[67.0;94.0] | 0.004 |
| HCT | 33.5[28.7;38.2] | 33.9[28.8;38.7] | 32.8[28.1;37.3] | 0.097 |
| Hb | 11.1[9.40;12.7] | 11.3[9.50;12.9] | 10.6[9.12;12.3] | 0.004 |
| PLT | 192[137;255] | 192[140;247] | 192[128;267] | 0.596 |
| RDW | 14.3[13.4;15.6] | 14.1[13.3;15.3] | 14.8[13.8;16.4] | <0.001 |
| RBC | 3.67[3.14;4.22] | 3.74[3.16;4.27] | 3.58[3.06;4.10] | 0.026 |
| WBC | 12.3[8.88;16.2] | 12.1[8.70;15.5] | 13.1[9.33;18.5] | <0.001 |
| ALB | 3.10[2.70;3.50] | 3.10[2.70;3.50] | 3.00[2.50;3.30] | <0.001 |
| AG | 15.0[12.0;17.0] | 14.0[12.0;17.0] | 15.0[13.0;18.8] | <0.001 |
| Glu | 142[115;183] | 138[114;179] | 153[117;197] | 0.006 |
| K | 4.00[3.70;4.50] | 4.00[3.70;4.50] | 4.10[3.80;4.70] | 0.003 |
| Na | 139[137;142] | 139[137;142] | 139[136;143] | 0.547 |
| CL | 105[101;109] | 105[101;109] | 106[100;109] | 0.395 |
| LAC | 1.90[1.30;3.00] | 1.90[1.30;2.90] | 2.04[1.36;3.33] | 0.010 |
| PCO2 | 40.0[34.0;46.0] | 40.0[35.0;46.0] | 40.0[34.0;48.0] | 0.440 |
| PO2 | 126[73.0;220] | 134[78.0;230] | 104[62.0;196] | <0.001 |
| INR | 1.30[1.10;1.50] | 1.20[1.10;1.50] | 1.30[1.20;1.70] | <0.001 |
| PT | 13.8[12.5;16.6] | 13.6[12.4;16.0] | 14.7[12.9;18.4] | <0.001 |
| PTT | 30.1[26.4;36.6] | 29.5[26.1;35.6] | 31.7[28.1;39.6] | <0.001 |
| ALT | 34.0[19.0;72.0] | 33.0[19.0;69.0] | 36.2[20.0;91.0] | 0.083 |
| AST | 49.0[28.0;116] | 46.0[27.0;104] | 57.0[31.0;155] | 0.002 |
| TB | 0.60[0.40;1.10] | 0.60[0.40;1.10] | 0.60[0.40;1.20] | 0.294 |
| CRE | 1.00[0.80;1.50] | 1.00[0.80;1.40] | 1.10[0.80;1.80] | <0.001 |
| URE | 19.0[13.0;31.0] | 18.0[13.0;27.8] | 23.0[16.0;37.0] | <0.001 |
| MV: |  |  |  | 0.232 |
| No | 244(19.9%) | 167(19.0%) | 77(22.3%) |  |
| Yes | 980(80.1%) | 711(81.0%) | 269(77.7%) |  |
| VP: |  |  |  | 0.795 |
| No | 349(28.5%) | 248(28.2%) | 101(29.2%) |  |
| Yes | 875(71.5%) | 630(71.8%) | 245(70.8%) |  |
| SA: |  |  |  | 0.096 |
| No | 97(7.92%) | 62(7.06%) | 35(10.1%) |  |
| Yes | 1127(92.1%) | 816(92.9%) | 311(89.9%) |  |
| CRRT: |  |  |  | 0.830 |
| No | 1102(90.0%) | 792(90.2%) | 310(89.6%) |  |
| Yes | 122(9.97%) | 86(9.79%) | 36(10.4%) |  |
