# Supplementary material for: The relationship between elderly nutritional risk index and short-term all-cause mortality in critically ill patients with cerebral injury: a retrospective cohort study from two cohorts
Source: Front Nutr. 2025 Jul 24;12:1620364. doi: 10.3389/fnut.2025.1620364 (PMC12328167; doi:10.3389/fnut.2025.1620364)
Supplement: Supplementary file 7 [file Table_3.docx]

TableS3: Summary descriptives table by groups of 28day all-cause mortality rate (External queue)

|  | **ALL** | **Survivor** | **No survivor** | **P overall** |
| --- | --- | --- | --- | --- |
|  | ***N=428*** | ***N=327*** | ***N=101*** |  |
| GNRI | 86.4 [80.4;92.3] | 87.9 [81.9;93.8] | 83.4 [77.4;87.9] | <0.001 |
| GNRI group: |  |  |  | <0.001 |
| No risk | 59 (13.8%) | 53 (16.2%) | 6 (5.94%) |  |
| Low risk | 57 (13.3%) | 53 (16.2%) | 4 (3.96%) |  |
| Moderate risk | 177 (41.4%) | 133 (40.7%) | 44 (43.6%) |  |
| High risk | 135 (31.5%) | 88 (26.9%) | 47 (46.5%) |  |
| Age | 68.0 [54.0;79.0] | 66.0 [52.0;77.5] | 73.0 [62.0;82.0] | <0.001 |
| Gender: |  |  |  | 0.925 |
| F | 153 (35.7%) | 116 (35.5%) | 37 (36.6%) |  |
| M | 275 (64.3%) | 211 (64.5%) | 64 (63.4%) |  |
| Weight | 81.0 [69.0;96.0] | 80.5 [68.9;96.0] | 82.2 [69.5;95.5] | 0.899 |
| Height | 170 [163;178] | 170 [163;178] | 170 [165;178] | 0.833 |
| BMI | 27.2 [24.3;31.9] | 27.2 [24.3;31.9] | 27.2 [24.2;31.6] | 0.755 |
| Hypertension: |  |  |  | 0.105 |
| No | 222 (51.9%) | 162 (49.5%) | 60 (59.4%) |  |
| Yes | 206 (48.1%) | 165 (50.5%) | 41 (40.6%) |  |
| AKI: |  |  |  | <0.001 |
| No | 216 (50.5%) | 181 (55.4%) | 35 (34.7%) |  |
| Yes | 212 (49.5%) | 146 (44.6%) | 66 (65.3%) |  |
| CKD: |  |  |  | 0.024 |
| No | 352 (82.2%) | 277 (84.7%) | 75 (74.3%) |  |
| Yes | 76 (17.8%) | 50 (15.3%) | 26 (25.7%) |  |
| Diabetes: |  |  |  | 0.439 |
| No | 291 (68.0%) | 226 (69.1%) | 65 (64.4%) |  |
| Yes | 137 (32.0%) | 101 (30.9%) | 36 (35.6%) |  |
| HF: |  |  |  | 0.212 |
| No | 323 (75.5%) | 252 (77.1%) | 71 (70.3%) |  |
| Yes | 105 (24.5%) | 75 (22.9%) | 30 (29.7%) |  |
| COPD: |  |  |  | 0.906 |
| No | 365 (85.3%) | 278 (85.0%) | 87 (86.1%) |  |
| Yes | 63 (14.7%) | 49 (15.0%) | 14 (13.9%) |  |
| SOFA | 7.00 [4.00;10.0] | 6.00 [3.00;9.00] | 10.0 [6.00;12.0] | <0.001 |
| APSII | 52.5 [37.0;75.0] | 48.0 [36.0;68.0] | 69.0 [45.0;113] | <0.001 |
| SAPII | 43.5 [32.0;56.0] | 42.0 [31.0;52.0] | 50.0 [35.0;73.0] | <0.001 |
| OASIS | 38.0 [31.0;44.0] | 37.0 [31.0;43.0] | 40.0 [34.0;48.0] | 0.002 |
| GCS | 15.0 [11.0;15.0] | 15.0 [11.0;15.0] | 15.0 [10.0;15.0] | 0.916 |
| HR | 86.5 [74.0;100] | 87.0 [74.0;100] | 86.0 [73.0;99.0] | 0.952 |
| RR | 19.0 [15.8;22.0] | 18.0 [15.0;22.0] | 19.0 [16.0;24.0] | 0.250 |
| NBPS | 125 [105;142] | 127 [107;142] | 119 [100;139] | 0.024 |
| NBPD | 67.0 [55.0;79.0] | 68.0 [56.0;80.0] | 65.0 [53.0;78.0] | 0.086 |
| NBPM | 82.0 [69.0;95.0] | 83.0 [70.0;95.0] | 79.0 [64.0;95.0] | 0.096 |
| HCT | 33.9 [28.4;37.9] | 34.3 [28.8;38.5] | 32.6 [27.4;37.1] | 0.086 |
| Hb | 11.2 [9.38;12.7] | 11.4 [9.50;13.0] | 10.7 [8.90;12.0] | 0.023 |
| PLT | 190 [134;241] | 191 [136;236] | 188 [125;267] | 0.441 |
| RDW | 14.3 [13.4;15.5] | 14.1 [13.3;15.1] | 14.8 [13.8;16.7] | 0.001 |
| RBC | 3.67 [3.14;4.21] | 3.75 [3.20;4.25] | 3.58 [2.96;3.95] | 0.032 |
| WBC | 12.4 [9.07;16.1] | 12.2 [8.75;15.7] | 14.2 [10.1;18.6] | 0.007 |
| ALB | 3.00 [2.70;3.40] | 3.10 [2.75;3.55] | 2.80 [2.40;3.10] | <0.001 |
| AG | 14.0 [12.8;17.0] | 14.0 [12.0;16.0] | 15.0 [13.0;18.0] | 0.023 |
| Glu | 142 [113;181] | 138 [113;180] | 150 [113;192] | 0.377 |
| K | 4.00 [3.60;4.50] | 3.90 [3.60;4.40] | 4.10 [3.80;4.70] | 0.070 |
| Na | 139 [136;142] | 140 [136;142] | 139 [136;142] | 0.640 |
| CL | 105 [100;109] | 105 [101;109] | 105 [98.0;109] | 0.200 |
| LAC | 1.80 [1.30;2.90] | 1.80 [1.25;2.70] | 2.10 [1.30;3.90] | 0.027 |
| PCO2 | 40.0 [35.0;45.0] | 39.4 [35.0;44.0] | 41.0 [33.0;51.0] | 0.203 |
| PO2 | 134 [78.0;223] | 143 [79.5;230] | 104 [72.0;185] | 0.027 |
| INR | 1.30 [1.10;1.60] | 1.30 [1.10;1.60] | 1.30 [1.10;1.60] | 0.876 |
| PT | 14.4 [12.6;17.7] | 14.1 [12.6;17.6] | 14.7 [12.7;17.8] | 0.318 |
| PTT | 31.0 [26.7;38.3] | 31.0 [26.6;38.6] | 30.9 [28.0;37.7] | 0.736 |
| ALT | 27.0 [16.0;57.0] | 25.0 [16.0;54.2] | 32.0 [16.0;74.0] | 0.086 |
| AST | 41.0 [25.0;92.2] | 39.0 [24.0;82.5] | 50.0 [26.0;130] | 0.032 |
| TB | 0.60 [0.40;1.10] | 0.60 [0.40;1.02] | 0.60 [0.40;1.46] | 0.623 |
| CRE | 1.05 [0.80;1.70] | 1.00 [0.72;1.50] | 1.50 [0.90;2.00] | <0.001 |
| URE | 19.0 [13.8;33.0] | 18.0 [13.0;28.0] | 28.0 [17.0;40.0] | <0.001 |
| VP: |  |  |  | 0.117 |
| No | 120 (28.0%) | 85 (26.0%) | 35 (34.7%) |  |
| Yes | 308 (72.0%) | 242 (74.0%) | 66 (65.3%) |  |
| Time | 8.88 [4.74;15.2] | 9.59 [4.72;16.0] | 8.41 [4.76;13.0] | 0.116 |
